# Long-term spatio-temporal precipitation variations in China with precipitation surface interpolated by ANUSPLIN

**DOI:** 10.1038/s41598-019-57078-3

**Published:** 2020-01-09

**Authors:** Binbin Guo, Jing Zhang, Xianyong Meng, Tingbao Xu, Yongyu Song

**Affiliations:** 1 Beijing Laboratory of Water Resources Security, Capital Normal University, Beijing, 100048 China; 2 Hunan Provincial Key Laboratory for Technology and Application of Cultural Heritage Digitalization, Hengyang Normal University, Hengyang, 421002 China; 3 College of Resources and Environmental Sciences, China Agricultural University (CAU), Beijing, 100094 China; 4 Department of Civil Engineering, The University of Hong Kong (HKU), Pokfulam, 999077 Hong Kong, China; 5 Fenner School of Environment and Society, The Australian National University, Canberra, ACT 2601 Australia

**Keywords:** Hydrology, Hydrology

## Abstract

Climate changes significantly impact environmental and hydrological processes. Precipitation is one of the most significant climatic parameters and its variability and trends have great influences on environmental and socioeconomic development. We investigate the spatio-temporal variability of precipitation occurrence frequency, mean precipitation depth, PVI and total precipitation in China based on long-term precipitation series from 1961 to 2015. As China’s topography is diverse and precipitation is affected by topography strongly, ANUSPLIN can model the effect of topography on precipitation effectively is adopted to generate the precipitation interpolation surface. Mann–Kendall trend analysis and simple linear regression was adopted to examine long-term trend for these indicators. The results indicate ANUSPLIN precipitation surface is reliable and the precipitation variation show different regional and seasonal trend. For example, there is a sporadic with decreasing frequency precipitation trend in spring and a uniform with increasing frequency trend in summer in Yangtze Plain, which may affect spring ploughing and alteration of flood risk for this main rice-production areas of China. In north-western China, there is a uniform with increasing precipitation frequency and intensity trend, which is beneficial for this arid region. Our study could be helpful for other counties with similar climate types.

## Introduction

Climate changes, including change in regional precipitation pattern, have modified hydrological processes and will continue to reshape the hydrology. Precipitation is an important meteorological parameter, and is a key player in regional hydrological processes^1,2^. It is an important natural source of water for vegetation, especially in dry regions, and its variability and trends have great influences on plant growth and crop production. Variation in total quantity and temporal variability of precipitation has significant effect on soil water supply, water stress as well as metabolic and physiological functions of vegetation, further determined the impact of precipitation variability on ecosystem structure^3^. It is of practical interest to access how climate changes have altered precipitation spatially and temporally and therefore to improve our insight into precipitation variability analyses.

Gauge-based precipitation is the main data source for precipitation. However, *in situ* precipitation data of rain gauge station are not always available. As precipitation measurements are scarce and the measuring stations are distributed unevenly in space, the rain gauge network might be inefficient in demonstrating the spatial distribution of precipitation and capturing precipitation events completely. Consequently, interpolation is introduced to examine the spatial distribution of precipitation.

To assess the spatial variability and trends of precipitation indicators, the procedures of spatial interpolation broadly included two strategies, interpolating of the station indices directly and interpolating of the precipitation accompanied by calculation of the indicator for every grid^4^. The first category first calculates the precipitation indices in each station. Interpolations of gauge-based precipitation indices can, therefore, generate a continuous map when analysing spatial differences. However, Rain gauge can only reflect the trend and variability of a small zone and there are data gaps between the gauges. The interpolation of station-based indices may be unable to reflect the accurate distribution of these indices. The second category is based on independently interpolated current rainfall grid surfaces. The generation of grid precipitation data can fill climate data gaps. It also facilitates the assessment of precipitation change impacts in un-surveyed areas, displaying consecutive precipitation surfaces and coupling them with climate models^5^.

There are many studies on the interpolation of precipitation though they differ in ease of use and effectiveness. The choice of which to select depends on the goals of the study and the territorial condition of the area (i.e., the topography and density of the precipitation observation network)^6^. Conventional interpolation approaches, including the Thiessen polygon method^7,8^ and inverse distance weighting method^9^, are widely adopted for precipitation interpolation^10^. While these approaches only suit for application over comparatively flat areas, since they assume that precipitation varies linearly between stations, they do not consider the effect of terrain on rainfall, which is one of the most significant factors influencing the distribution of rainfall. Precipitation is strongly affected by topography. The ANUSPLIN^11^ (short for Australian National University Spline) can model the effect of topography effectively as it involves both the horizontal and vertical coordinate system while interpolating precipitation. ANUSPLIN based on thin plate spline technique, has been used in many studies^12,13^ and has proved to be reliable for precipitation interpolation^14^. Ordinary kriging^15^ belong to geostatistical technique demanding prior calibration for its parameters by a semivariogram. The advantages of the ANUSPLIN package over kriging include its simplicity and a separate prior calibration for its parameters is not needed.

A number of interpolated grid climatic data sets (such as the WorldClim1^13^, WorldClim2^16^ APHRODITE^17^, and EA05^18^, etc.) were developed around the world. ANUSPLIN contributes to the construction of many climatic datasets and many of them are widely used around the world. For example, Mark *et al*.^19,20^ developed the monthly Climatic Research Unit (CRU) data set (including 8 climate elements) with ANUSPLIN over global land area (except for Antarctica). Hijmans *et al*.^13^ developed the monthly WorldClim 1 data set (period from 1960 to 1990), and Fick and Hijmans^16^ developed and updated WorldClim 2 data set (period from 1970 to 2000) for global land areas (excluding Antarctica) with ANUSPLIN. Specific to China, Xu *et al*.^21^ constructed the daily temperature data set named CN05 (period from 1961 to 2005, resolution of 0.5°) with ANUSPLIN over mainland China. Comparing to CN05, the CN05.1^22^ (including both temperature and precipitation dataset) is generated with ANUSPLIN using a lot of data from extra observing stations. Hong *et al*.^23^ generated monthly gridded mean temperature and precipitation datasets (resolution of 0.01°) using ANUSPLIN. The daily gridded precipitation dataset (resolution of 0.5°) developed by the China Meteorological Administration is also interpolated with ANUSPLIN^24^. However, the temporal-spatial resolution of these datasets are coarse and high temporal-spatial datasets are necessary for examining the trends and variability of precipitation in China.

A variety of studies have been proposed to explore the variability of precipitation in China^25–27^. Zhang^28^ found significant downward trends of precipitation frequency and significant upward trends of precipitation intensity over China for the period 1956–2005. Zhai^29^ reported no trend of total precipitation for China in general, and the precipitation frequency reduced significantly within most areas of China except the northwestern region. Liu^30^ found that precipitation increased; meanwhile, the precipitation frequency decreased in China from 1960 to 2000. However, most of these studies are either belong to category 1) direct interpolation from the station precipitation indices, or category 2) conventional interpolation of gauge-based precipitation accompanied by calculation of the index at each grid. China’s topography is varied and complex and precipitation is strongly affected by topography, while conventional precipitation interpolation methods underestimate the effect of terrain on precipitation.

In this study, based on long-term (1961–2015) daily (monthly) precipitation data from 819 (756) stations in China, the ANUSPLIN is used to generate the precipitation interpolation surface with high spatio-temporal resolution. This study intend to synthetically evaluate the spatio-temporal variability of precipitation with high resolution data so as to ameliorate the understanding of the precipitation variability in China. We estimated the spatio-temporal variability of precipitation occurrence frequency, mean precipitation depth, precipitation variability index (PVI) and total precipitation in China based on the generated precipitation interpolation surface. Precipitation occurrence frequency, mean precipitation depth, and total precipitation describe the frequency, intensity, and amount of precipitation respectively. PVI is a new dimensionless indicators and is put forward recent years. It has been recommended as a potential key indicator to measure the ecological impacts of rainfall patterns on vegetation under the changing climate^3^. The spatio-temporal variability of these precipitation indicators was obtained by calculating the indicators for each grid. This research provides more insight into precipitation variability across China.

China is a very large country with a variety of climates and environments. The diverse climate span from arid region in the northwest part to alpine region in the Tibetan Plateau and monsoon region in the east and south part. The monsoon region include tropical in the southern part to humid in the eastern part and is the main grain producing area. As many other countries in the world experience similar climate types and environments, our study in China under climate change could provide valuable information such as water resources management, agricultural management and relative adaptation strategies for other counties.

## Results

In the study, we investigated four precipitation indicators depicting the frequency, intensity, variability and amount of precipitation with precipitation occurrence frequency (λ_0_), mean precipitation depth (1/θ) and precipitation variability index (PVI) and total precipitation (TP) in China. For accessing the temporal variability of these indicators, the precipitation indicators were calculated within certain periods and these were defined as a year or a season, i.e. dry season (January–April, November–December), wet season (May–October), spring (March–May), summer (June–August), autumn (September–November), and winter (December–February). In order to explore the spatial distribution of these indicators and facilitate the description of changes in regional precipitation characteristics, China was divided into six sub-regions, namely: northeast (NE), north China plain (NCP), southwest (SW), southeast (SE), Qinghai-Tibet Plateau (QP) and northwest (NW).

**Figure 1:**
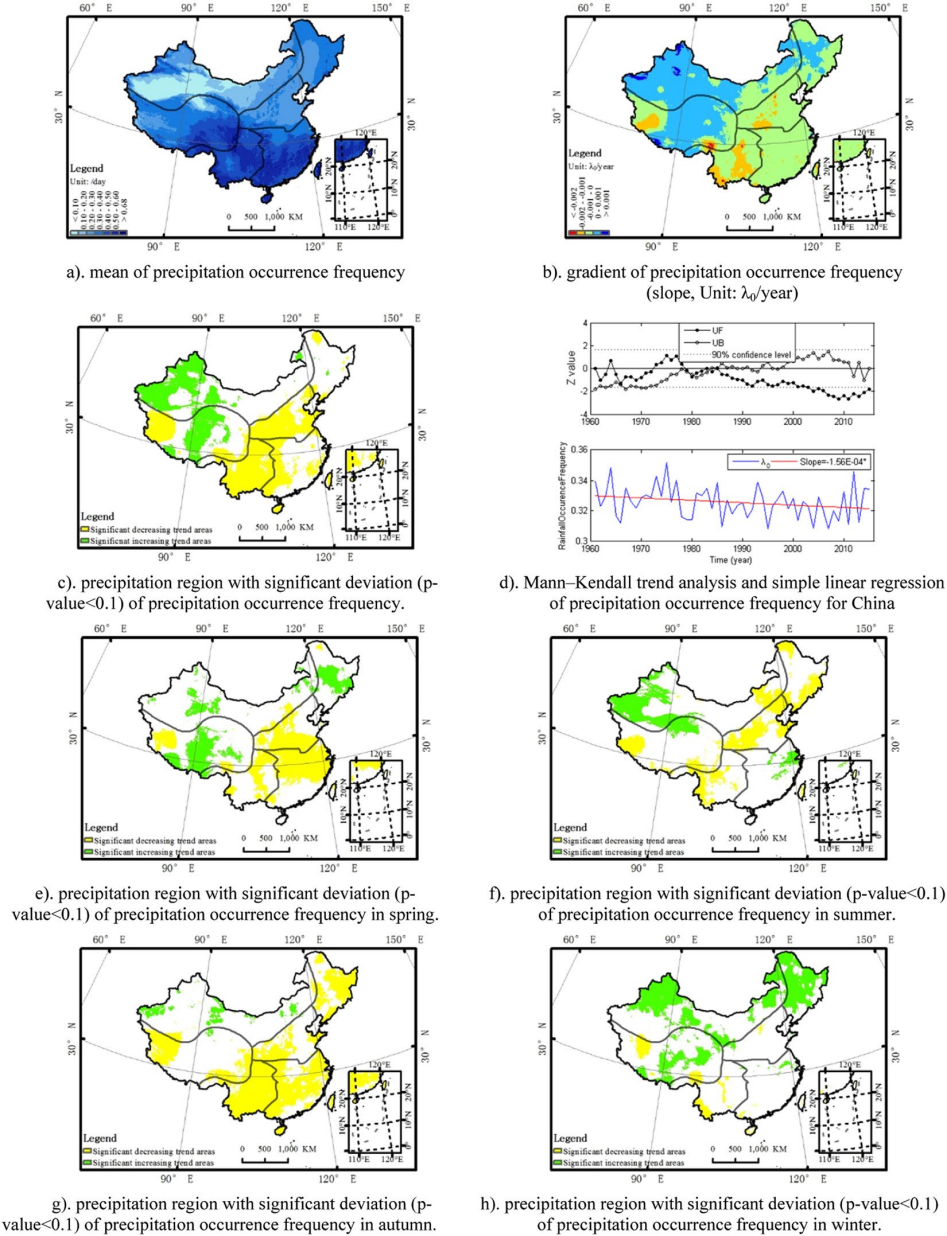
The annual spatio-temporal distribution of precipitation occurrence frequency (λ_0_) in China (1961–2015). (**a**) mean of precipitation occurrence frequency. (**b**) gradient of precipitation occurrence frequency (slope, Unit: λ_0_/year). (**c**) precipitation region with significant deviation (p-value < 0.1) of precipitation occurrence frequency. (**d**) Mann–Kendall trend analysis and simple linear regression of precipitation occurrence frequency for China. (e) precipitation region with significant deviation (p-value < 0.1) of precipitation occurrence frequency in spring. (**f**) precipitation region with significant deviation (p-value < 0.1) of precipitation occurrence frequency in summer. (**g**) precipitation region with significant deviation (p-value < 0.1) of precipitation occurrence frequency in autumn. (**h**) precipitation region with significant deviation (p-value < 0.1) of precipitation occurrence frequency in winter.

Precipitation occurrence frequency describes the frequency of precipitation and is denoted as the count of rainy days divide by the amount of days in a certain period. Generally, the precipitation occurrence frequency in the south-east was larger than that in the north-west. Precipitation occurrence frequency decreased significantly in all China, with significant regional difference between south-east and north-west. The frequency of precipitation showed a significant downward trend in the south-east region while upward trend in north-west region. In north-west region, the positive trend of precipitation occurrence frequency was significant during the dry season. In the center of Qinghai-Tibet Plateau region, the precipitation occurrence frequency showed a significant upward trend; while in the west of Qinghai-Tibet Plateau region, there was a significant negative trend of precipitation occurrence frequency (Fig. 1c). Seasonally, there was a significant increasing trend of precipitation occurrence frequency in the northwest, Qinghai-Tibet Plateau and northeast regions while a significant trend of decrease in the southwest area for the dry season. The precipitation occurrence frequency showed a significant downward trend in northeast, north China plain, southwest and southeast region for the wet season. There was a significant positive trend of precipitation occurrence frequency in northwest and northeast region in winter. A Mann–Kendall trend analysis shows that there is a breakpoint around 1980s for the precipitation occurrence frequency in China. The spatial distribution of the mean, deviation, region with significant deviation and a Mann–Kendall trend analysis and simple linear regression plot for precipitation occurrence frequency at an annual time scale (Fig. 1a–d) and region with significant deviation for each season (Fig. 1e–h) is described as the figures below.

**Figure 2:**
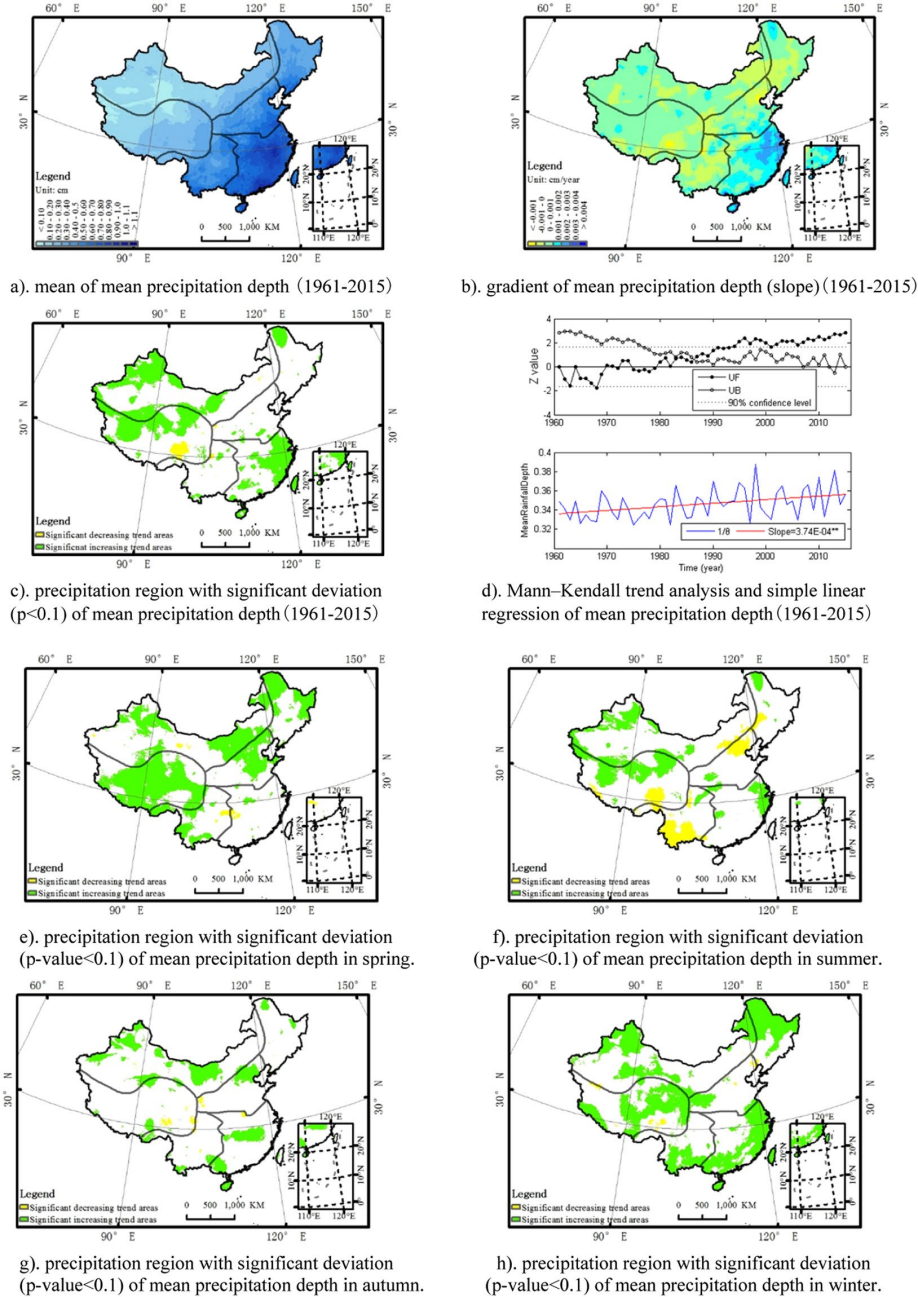
The annual spatio-temporal distribution of mean precipitation depth (1/θ) in China. The map was generated by the software ArcGIS 10.1. (**a**) mean of mean precipitation depth (1961–2015). (**b**). gradient of mean precipitation depth (slope)(1961–2015). (**c**) precipitation region with significant deviation (p < 0.1) of mean precipitation depth(1961–2015). (**d**) Mann–Kendall trend analysis and simple linear regression of mean precipitation depth(1961–2015). (**e**) precipitation region with significant deviation (p-value < 0.1) of mean precipitation depth in spring. (**f**) precipitation region with significant deviation (p-value < 0.1) of mean precipitation depth in summer. (**g**) precipitation region with significant deviation (p-value < 0.1) of mean precipitation depth in autumn. (**h**) precipitation region with significant deviation (p-value < 0.1) of mean precipitation depth in winter.

The precipitation depth (unit: centimetre) describes the rainfall amount of a rainfall event. The mean precipitation depth denoted as the total rainfall amount divide by the count of rainy days in a certain period is an index used to describe the precipitation intensity. The mean precipitation depth in the southeast was larger than that in the northwest. Overall, there was a significant increase trend of mean precipitation depth in China. Regionally, the intensity of precipitation shows significant upward trend in southeast region and in the north region of northwest and Qinghai-Tibet Plateau, though there is a significant downward trend in the Lancang River headwaters area located in the south region of Qinghai-Tibet Plateau. Seasonally, the mean precipitation depth in the north of the north China plain, the southwest of the southwest China, and Lancang River headwaters area shows a significant decrease trend in the summer. A Mann–Kendall trend analysis of mean precipitation depth shows that there is a breakpoint around 1980s for the mean precipitation depth in China. The spatial distribution of the mean, deviation, region with significant deviation and the Mann–Kendall test and simple linear regression plot for mean precipitation depth at an annual time scale (Fig. 2a–d) and region with significant deviation for each season (Fig. 2e–h) is described as the figures below.

**Figure 3:**
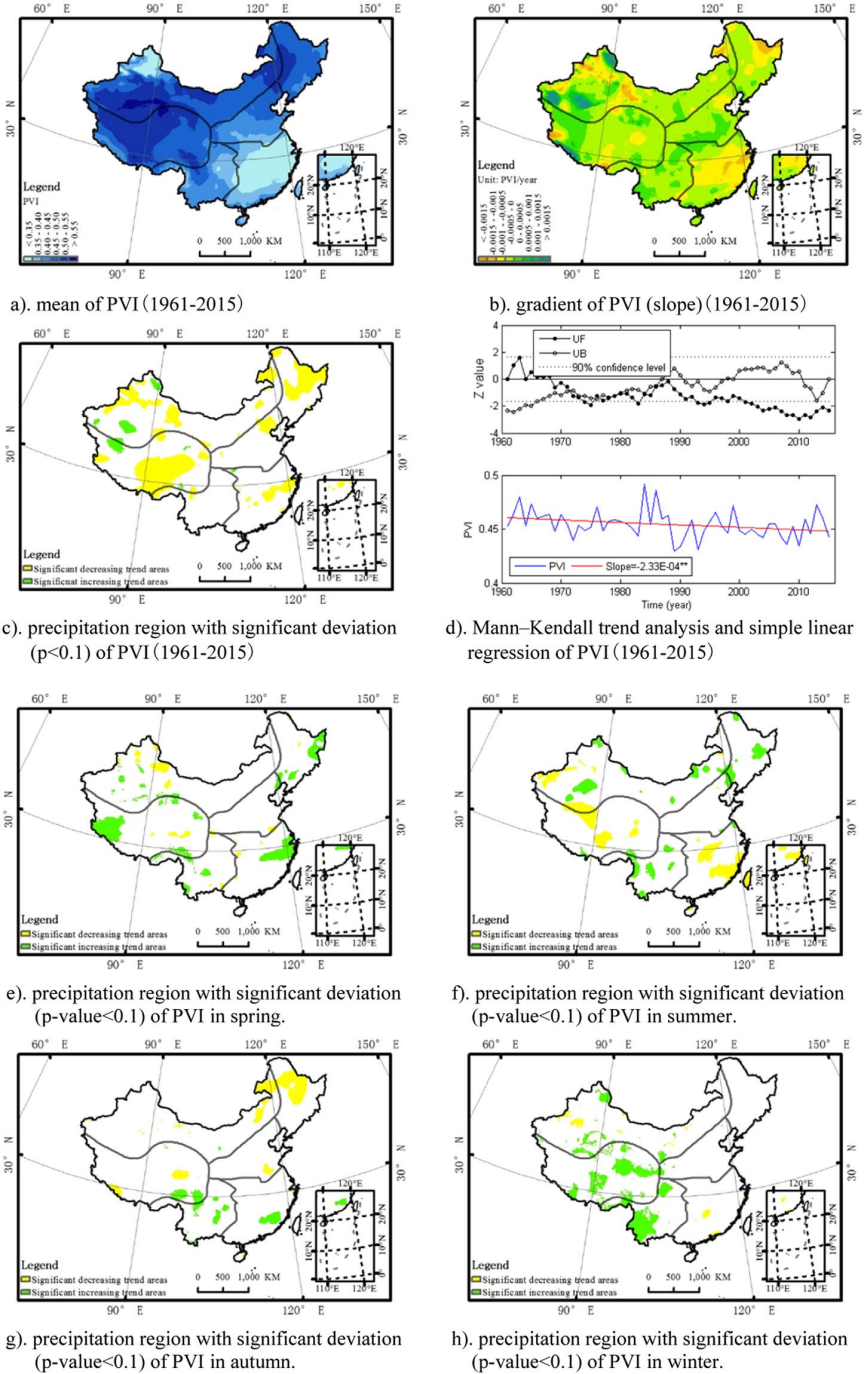
The annual spatio-temporal distribution of the PVI in China. The map was generated by the software ArcGIS 10.1. (**a**) mean of PVI(1961–2015). (**b**) gradient of PVI (slope)(1961–2015). (c) precipitation region with significant deviation (p < 0.1) of PVI(1961–2015). (**d**) Mann–Kendall trend analysis and simple linear regression of PVI(1961–2015). (**e**) precipitation region with significant deviation (p-value < 0.1) of PVI in spring. (**f**) precipitation region with significant deviation (p-value < 0.1) of PVI in summer. (**g**) precipitation region with significant deviation (p-value < 0.1) of PVI in autumn. (**h**) precipitation region with significant deviation (p-value < 0.1) of PVI in winter.

The PVI is a dimensionless index with a value approaching to zero for precisely consistent precipitation and increases as precipitation events get more infrequent. The spatial distribution for the annual average of PVI shows that the PVI in the northwest, Qinghai-Tibet Plateau and south of northeast was larger than that in the southeast. Precipitation in the southeast region and the Junggar Basin (located in the north of Tianshan Mountains, north of the northwest region) was more uniform than other regions, while precipitation in Qinghai-Tibet Plateau, north China plain and south region of northwest depicted a very sporadic and concentrated pattern (Fig. 3a).

**Figure 4:**
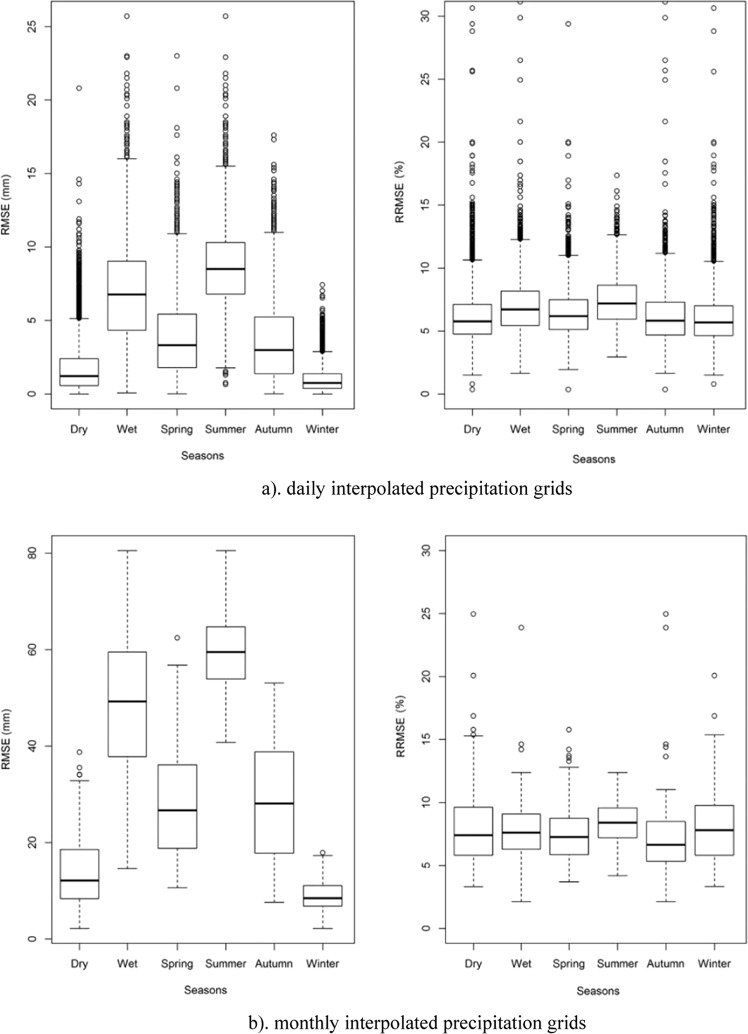
The error bars of average RMSE (unit: mm) and RRMSE (unit: %) in each season for daily and monthly interpolated precipitation grids (1961–2015). (**a**) daily interpolated precipitation grids. (**b**) monthly interpolated precipitation grids.

Overall, there was a similar significant pattern of decrease in PVI in China (Fig. 3b–d), which means there was a uniform trend in precipitation in all China, but the trend shows significant regional and seasonal difference. The PVI showed a significant negative trend in Northeast Plain-Xiaoxing an Mountains region (located in the northeast of China) and centre of Qinghai-Tibet Plateau. In the centre of Tarim Basin (located in the southwest of northwest region, China), southwest region of West Kunlun Mountains (located near the northwest of Qinghai-Tibet Plateau) and northeast region of Junggar Basin, there was a significant positive trend of PVI (Fig. 4c), which mean the precipitation events there become sporadic. Seasonally, the downward trend of PVI mainly happened during the summer and autumn, while there was an upward trend of PVI during the spring and winter. PVI showed a significant trend of increase in spring (Fig. 3e) while a significant trend of decrease in summer (Fig. 3f) in Yangtze Plain, which mean the precipitation pattern has a sporadic trend in spring, but a uniform trend in summer there. In autumn of the north-eastern Inner Mongolia and the northeast region, there is a uniform of precipitation as PVI depicted a significant downward trend (Fig. 3g). In summer (Fig. 3f) and winter (Fig. 3h) of the southwest region, there is a sporadic trend of precipitation as the PVI depicts a significant positive trend. PVI also indicated a significant upward trend in spring (Fig. 3e) and winter (Fig. 3h) in the Qinghai-Tibet Plateau.

The spatial distribution of the mean, deviation, region with significant deviation and a Mann–Kendall trend analysis and simple linear regression plot for PVI at an annual time scale (Fig. 3a–d) and region with significant deviation for each season (Fig. 3e–h) is described as the figures below. A Mann–Kendall trend analysis showed that the trend of PVI was locally unstable.

**Figure 5:**
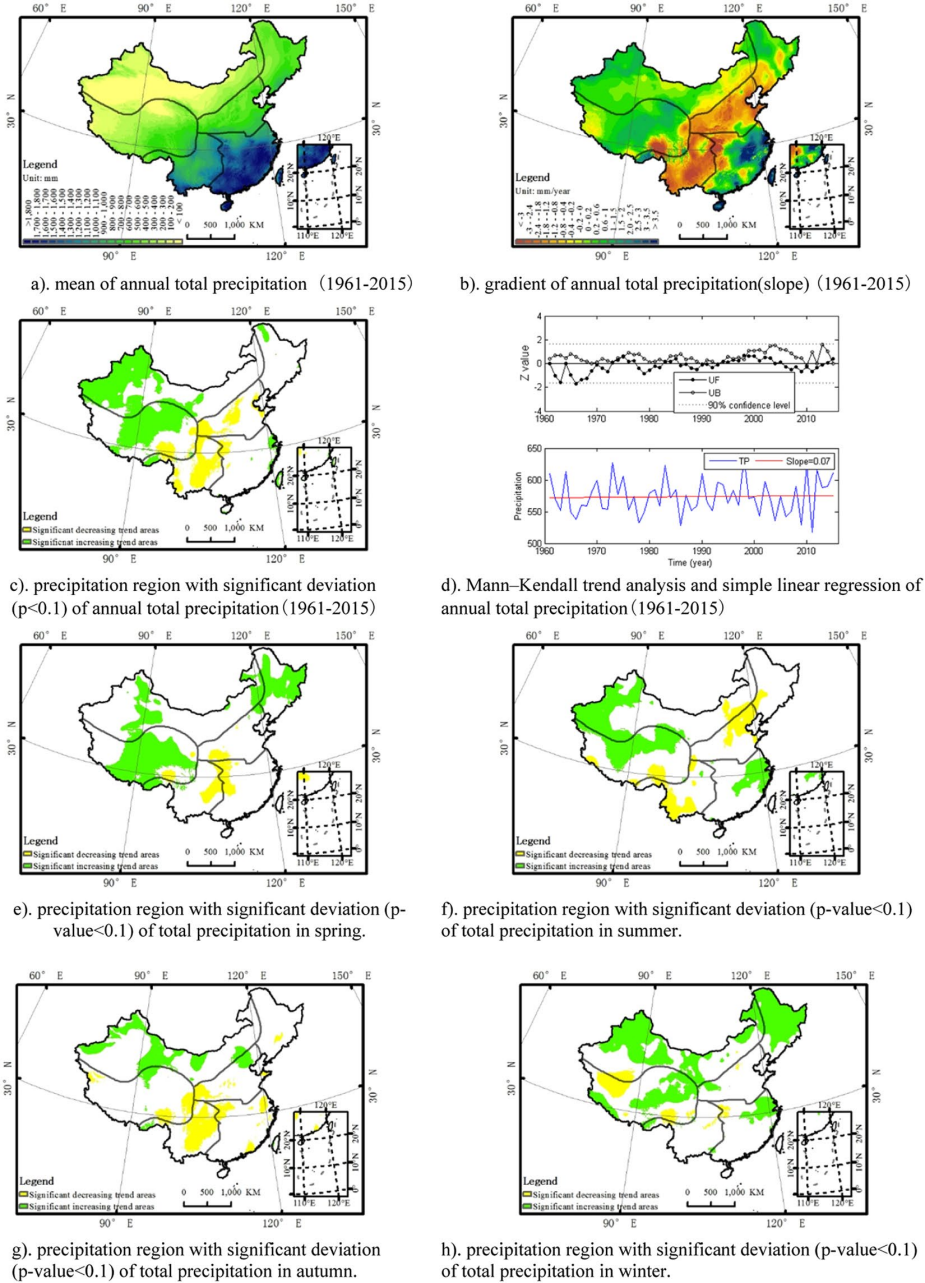
The annual spatio-temporal distribution of total precipitation in China. The map was generated by the software ArcGIS 10.1. (**a**) mean of annual total precipitation (1961–2015). (**b**) gradient of annual total precipitation(slope) (1961–2015). (**c**) precipitation region with significant deviation (p < 0.1) of annual total precipitation(1961–2015). (**d**) Mann–Kendall trend analysis and simple linear regression of annual total precipitation(1961–2015). (**e**) precipitation region with significant deviation (p-value < 0.1) of total precipitation in spring. (**f**) precipitation region with significant deviation (p-value < 0.1) of total precipitation in summer. (**g**) precipitation region with significant deviation (p-value < 0.1) of total precipitation in autumn. (**h**) precipitation region with significant deviation (p-value < 0.1) of total precipitation in winter.

Total precipitation is defined as the accumulation of precipitation within a period. The total precipitation in the southern China was greater than that in the northern China. The annual total precipitation showed a small upward trend (0.07 mm/yr) in all China with significant regional and seasonal difference. Regionally, there is an increasing total precipitation trend in northwest region (0.34 mm/yr), Qinghai-Tibet Plateau (0.34 mm/yr), northeast region (0.23 mm/yr), and southeast region(0.94 mm/yr), while there is a significant trend of increase in southwest region (−1.54 mm/yr) and north China plain (−1.21 mm/yr). Seasonally, in dry season, there is a significant upward trend of total precipitation in northwest region (0.14 mm/yr), Qinghai-Tibet Plateau region (0.12 mm/yr), and northeast (0.41 mm/yr) region, an positive trend in southeast region (0.3 mm/yr), while a trend of decrease in north China plain (−0.22 mm/yr) region and southwest region (−0.25 mm/yr). In wet season, there is a significant downward trend of total precipitation in north China plain (−0.99 mm/yr) and southwest region (−1.3 mm/yr), a decreasing trend in northeast region (−0.18 mm/yr), and an increasing trend in northwest region (0.2 mm/yr), Qinghai-Tibet Plateau (0.22 mm/yr), and southeast region (0.63 mm/yr). The total precipitation showed a significant trend of increase in northwest, Qinghai-Tibet Plateau and northeast region of China in spring, a significant increasing trend in southeast region while a significant decreasing trend in north China plain in summer, a significant increasing trend in northwest region while a significant decreasing trend in southwest region in autumn, and a significant increasing trend in northwest, Qinghai-Tibet Plateau and northeast region in winter. The spatial distribution of the mean, deviation, region with significant deviation and a Mann–Kendall trend analysis and simple linear regression plot for total precipitation at an annual time scale (Fig. 5a–d) and region with significant deviation for each season (Fig. 5e–h) is described as the figures below. A Mann–Kendall trend analysis revealed that the trend was locally unstable.

Figures 1–5 depicts the spatial distribution of the mean, deviation, region with significant deviation and the Mann–Kendall test and simple linear regression plot for each precipitation indicator at an annual time scale as well as region with significant deviation for each season. The simple linear regression slope of each precipitation indicator annually, seasonally and regionally are described as the table below (Table 1).

**Table 1:** The simple linear regression slope of precipitation indicators during the period 1961–2015. Note:* represents significance level at p < 0.1 and ** represents significance level at p < 0.05.

		China	NW	QP	NE	NCP	SW	SE
**λ**_**0**_	Annual	−1.56E-04*	2.44E-04**	5.00E-06	−1.10E-04	−6.17E-04**	−8.47E-04**	−5.31E-04**
	Dry season	7.90E-05	3.28E-04**	2.49E-04*	5.59E-04**	−3.47E-04	−6.04E-04**	−3.58E-04
	Wet season	−3.89E-04**	1.62E-04	−2.36E-04	−7.72E-04**	−8.84E-04**	−1.09E-03**	−7.02E-04*
	Spring	−1.17E-04	1.21E-04	3.87E-04	4.10E-04	−8.32E-04**	−5.93E-04*	−1.02E-03**
	Summer	−1.62E-04	2.34E-04	−1.20E-04	−8.31E-04**	−9.56E-04**	−5.22E-04	2.71E-04
	Autumn	−6.21E-04**	1.32E-04	−4.48E-04	−8.02E-04**	−8.00E-04*	−1.92E-03**	−1.41E-03**
	Winter	2.75E-04*	4.93E-04**	1.99E-04	7.86E-04**	1.24E-04	−3.57E-04	2.50E-05
**1/θ**	Annual	3.74E-04**	2.26E-04**	2.06E-04**	2.44E-04	2.56E-04	9.30E-05	1.36E-03**
	Dry season	4.92E-04**	2.53E-04*	3.31E-04**	6.14E-04*	2.60E-04	6.48E-04*	1.24E-03**
	Wet season	4.03E-04**	2.54E-04*	2.26E-04**	4.54E-04	2.51E-04	−3.70E-05	1.39E-03**
	Spring	8.07E-04**	6.00E-04**	5.76E-04**	1.16E-03**	1.58E-03**	7.57E-04*	7.91E-04
	Summer	2.17E-04	8.20E-05	1.33E-04	1.42E-04	−1.24E-04	−6.14E-04	1.55E-03**
	Autumn	5.74E-04**	5.66E-04**	2.16E-04	5.00E-04	4.77E-04	4.04E-04	1.39E-03**
	Winter	6.93E-04**	3.47E-04**	4.73E-04**	6.28E-04**	3.00E-05	1.13E-03**	2.06E-03**
**PVI**	Annual	−2.33E-04**	−1.81E-04	−1.40E-04	−4.36E-04**	−1.45E-04	−1.34E-04	−4.58E-04
	Dry season	7.98E-04	1.26E-03	6.64E-04	1.54E-03	4.59E-04	1.34E-03**	−7.83E-04
	Wet season	−3.93E-04*	−5.41E-04	−4.51E-04	−1.10E-04	−4.15E-04	2.27E-04	−6.20E-04
	Spring	1.51E-04	−1.38E-04	9.91E-04**	2.82E-04	−8.97E-04	4.35E-04	−2.10E-05
	Summer	−2.06E-04	−4.80E-05	−3.75E-04	4.93E-04	−3.90E-05	7.08E-04*	−1.64E-03**
	Autumn	−1.67E-03	−3.06E-03	−1.10E-05	−6.06E-03**	−3.11E-03	1.96E-03	1.09E-03
	Winter	2.24E-03*	1.93E-03	4.83E-03*	1.77E-03	7.84E-04	3.93E-03**	−7.65E-04
**TP**	Annual	0.07	0.34**	0.34*	0.23	−1.21*	−1.54**	0.94
	Dry season	0.11	0.14**	0.12**	0.41**	−0.22	−0.25	0.3
	Wet season	−0.05	0.2	0.22	−0.18	−0.99*	−1.30**	0.63
	Spring	0.03	0.10*	0.21**	0.44**	−0.13	−0.07	−0.56
	Summer	0.08	0.06	0.14	−0.27	−0.77*	−0.72	1.48**
	Autumn	−0.17	0.11**	−0.04	−0.12	−0.33	−0.79**	−0.5
	Winter	0.13**	0.07**	0.04*	0.18**	0.03	0.04	0.51

### ANUSPLIN validation

ANUSPLIN was applied to convert the original station-based precipitation data into precipitation surface grids. The precipitation indicators surface were calculated through the ANUSPLIN precipitation surfaces. The interpolation using ANUSPLIN enabled a robust detection of error on the precipitation surface grids by cross validation. The cross-validation statistics were used to evaluate the overall predictive error of the fitted spline surface in this study. The Mean Error, root mean square error, mean absolute error and relative root mean square error of these individual unweighted differences can be used to evaluate the overall predictive error of the fitted spline surface. The ME, RMSE, MAE and RRMSE (Table 2) as well as error bars for RMSE and RRMSE (Fig. 4) for these individual unweighted residuals are shown below.

**Table 2:** Mean Error (ME, unit: mm), Mean absolute error (MAE, unit: mm), root mean square error (RMSE, unit: mm) and Relative RMSE (RRMSE, unit: %) of daily and monthly precipitation interpolation surfaces through cross-validation.

		Annual	Dry	Wet	Spring	Summer	Autumn	Winter
**ME (unit: mm)**	Daily data set	−0.48	−0.17	−0.79	−0.40	−1.07	−0.36	−0.09
	Monthly data set	−2.09	−1.09	−3.09	−1.88	−3.82	−1.93	−0.73
**RMSE (unit: mm)**	Daily data set	4.30	1.75	6.81	3.88	8.66	3.59	1.01
	Monthly data set	31.17	14.23	48.12	28.06	59.57	28.09	8.97
**MAE (unit: mm)**	Daily data set	1.45	0.54	2.35	1.26	3.15	1.07	0.30
	Monthly data set	18.48	7.51	29.45	15.74	38.07	15.57	4.55
**RRMSE (unit: %)**	Daily data set	6.76%	6.30%	7.21%	6.60%	7.39%	6.67%	6.37%
	Monthly data set	8.21%	8.28%	8.13%	7.60%	8.63%	8.07%	8.52%

For the daily interpolated precipitation grid, the mean ME was −0.48 mm, the mean MAE was 1.45 mm, and the mean RMSE was 4.30 mm. For the monthly precipitation interpolation grid surface, the mean ME was −2.09 mm, the mean MAE was 18.48 mm, and the mean RMSE was 31.17 mm. The mean RMSE for the daily interpolated precipitation grid was 1.75 mm for the dry season and 6.81 mm for the wet season, and for monthly precipitation interpolation grid was 14.23 mm for the dry season and 48.12 mm for the wet season. As the magnitude of precipitation varies for different seasons, the RMSE (or MAE) of the interpolated grids is big during the wet season and small during the dry season, but the RRMSE was stable. The annual average error of the RRMSE was within 6.76% for daily precipitation (8.21% for monthly precipitation), which prove that ANUSPLIN precipitation surface is reliable for the calculation and analysis of indicators in China.

## Discussion

**Figure 6:**
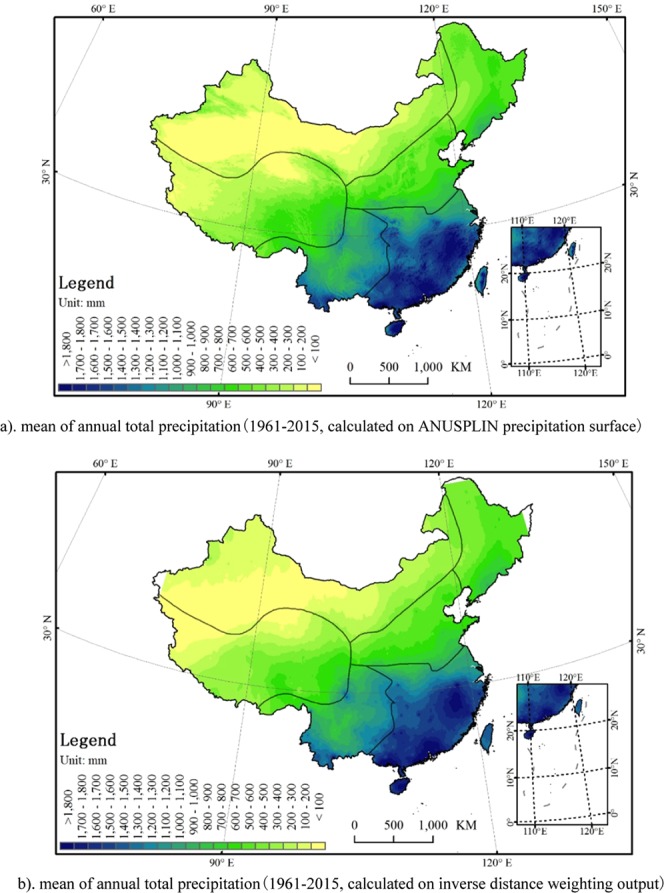
Mean of annual total precipitation calculated on ANUSPLIN precipitation surface (**a**) and inverse distance weighting output (**b**) in China (1961–2015). (**a**) mean of annual total precipitation (1961–2015, calculated on ANUSPLIN precipitation surface). (**b**) mean of annual total precipitation (1961–2015, calculated on inverse distance weighting output).

This study presents the long-term (1961 to 2015) spatio-temporal variations of precipitation in China with precipitation surface interpolated by ANUSPLIN. Precipitation is greatly affected by topography and the terrain in China is diverse. ANUSPLIN considering the effect of terrain can generate reliable precipitation grid surface. Although the general spatial distributions of many precipitation indicators generated from ANUSPLIN precipitation grids and conventional interpolation (i.e. inverse distance weighting) output are similar in China, the ANUSPLIN precipitation grid gives much more accurate distribution information for precipitation. For example, the annual precipitation map in China (1961–2015) generated using the ANUSPLIN precipitation surface (Fig. 6a) describe more reasonable precipitation distribution than the map generated using conventional inverse distance weighting method where there are a lot of sites distributed with a fake ring shape or parallel strip shape (Fig. 6b).

**Figure 7:**
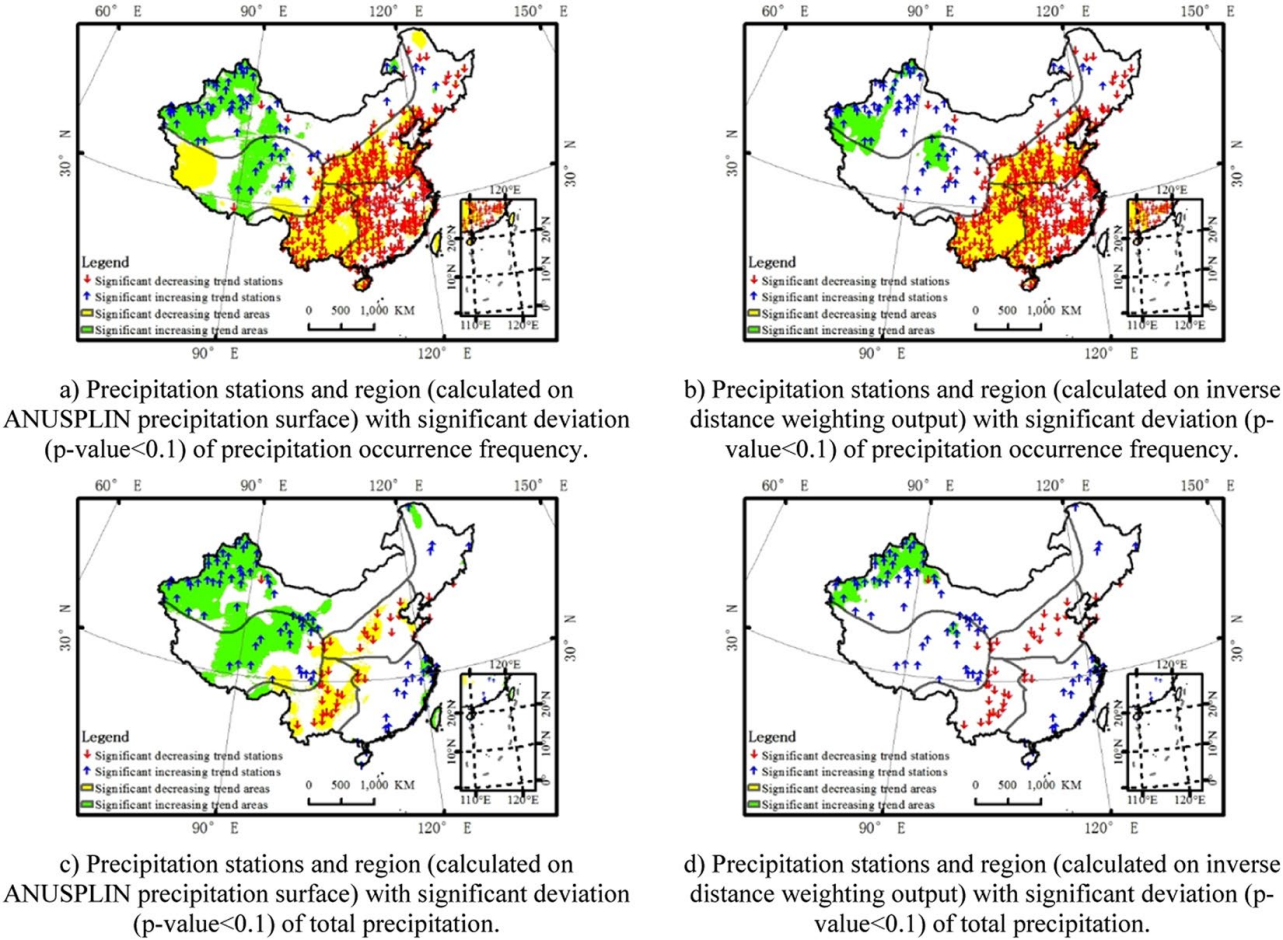
Precipitation stations and region with significant deviation (p-value < 0.1) of precipitation occurrence frequency and total precipitation in China (1961–2015) calculated on ANUSPLIN precipitation surface and inverse distance weighting output. (**a**) Precipitation stations and region (calculated on ANUSPLIN precipitation surface) with significant deviation (p-value < 0.1) of precipitation occurrence frequency. (**b**) Precipitation stations and region (calculated on inverse distance weighting output) with significant deviation (p-value < 0.1) of precipitation occurrence frequency. (**c**) Precipitation stations and region (calculated on ANUSPLIN precipitation surface) with significant deviation (p-value < 0.1) of total precipitation. (**d**) Precipitation stations and region (calculated on inverse distance weighting output) with significant deviation (p-value < 0.1) of total precipitation.

ANUSPLIN show its priority to other interpolation method in modelling the terrain effect on precipitation. Comparing to conventional inverse distance weighting method, the ANUSPLIN gives much more details in precipitation variation, especially for areas where the topography is various. Taking the precipitation indicator precipitation occurrence frequency and total precipitation for examples, comparing the map of significant deviation region for precipitation indicators generated from ANUSPLIN precipitation grids (Fig. 7a,c) and inverse distance weighting output (Fig. 7b,d), many areas with significant trend are caught by ANUSPLIN precipitation surface while missed for inverse distance weighting output, especially in the  central and western regions where the terrain is complicated. Comparing to gauge precipitation, ANUSPLIN precipitation surface also show its priority. More information was available with the analysis based on the spatially distributed ANUSPLIN precipitation grid surface than with station-based precipitation, especially for data-scarce regions. For example, there are many regions with significant downward (or upward) trend of precipitation occurrence frequency and total precipitation in western China (Fig. 7a,c), which was caught by ANUSPLIN precipitation grid surface while not caught by observation stations.

Using the precipitation grid surface, the spatio-temporal analysis of precipitation occurrence frequency, mean precipitation depth and total precipitation depicts a significant decreasing trend of precipitation frequency, significant increasing trends of precipitation intensity, and a small upward trend in annual total precipitation over China, which is consistent with the result from previous researches at a national scale^29–31^. Though the results from previous studies showed consistent varying tendency and similar spatial distributions with the results in this work, there are a lot of discrepancies exists in the quantitative analysis and spatial distributions of rainfall. The difference might come from the spatial difference of precipitation from different interpolation methods. The precipitation interpolation performance is one of the principal uncertainty source for precipitation spatio-temporal variability analysis. Developing gridding precipitation indices from daily precipitation observations is not forthright since averaging daily precipitation information from numerous stations and interpolating it into gridded precipitation data dampens the extremes and misrepresents the spatial and temporal variability that exists in the original station data^5^. If the rain gauges are extremely sparse, acquiring a correct distribution of rainfall through interpolation is even more difficult and the low density of precipitation stations leads to significant uncertainties, especially in regions where the rainfall varies significantly in space and time^32–34^. Comparing to conventional interpolation method, the ANUSPLIN show its priority in modelling the precipitation under complex terrain and data-scarce condition. This study may provide a more reasonable result and depict more accurate spatio-temporal distribution of precipitation indicators in China since it is calculated based on reliable ANUSPLIN precipitation surface grid.

As daily precipitation has a particular stochastic nature, daily precipitation shows different pattern to monthly precipitation^35^. The aggregation of a daily interpolated precipitation grid to a monthly precipitation grid may also accumulate interpolation errors from the daily precipitation surface. We recommend that the monthly precipitation grid is formed through interpolation from monthly station-based precipitation. This is why indices such as total precipitation were calculated based on the monthly interpolated grid surface while other indices were based on the daily interpolated grid surface. In this study, precipitation indicators such as precipitation occurrence frequency, mean precipitation depth, and PVI were based on the interval of daily precipitation events. However, due to the limitation of the dataset, the resolution of this interval was limited to one daily measurement. These indices cannot separate the precipitation intervals at timescales smaller than one day. More specific information or higher time resolution precipitation dataset is needed for further elaborate precipitation variability studies. Moreover, different partitioning methods may result in uncertainties in describing regional features of precipitation for the regional analysis. This division follows a commonly used agriclimatic regionalization of China dividing China into northwest, Qinghai-Tibet Plateau and monsoon regions^36^, and then dividing monsoon regions into southeast, southwest, north China plain and northeast^37^.

Precipitation is an important natural source of water for vegetation and agriculture. The temporal variability of precipitation has important effect on soil water supply, water stress as well as metabolic and physiological functions of vegetation. The variability and trends of precipitation have significant impacts on crop production and plant growth. PVI, a new indicators that has been put forward recent years, is recommended as a potential key indicator to quantify the temporal variation of precipitation patterns and access its impacts on plant (i.e. effects of drought on tree mortality) as well as Eco hydrology under the changing climate. PVI has not been used in detecting the precipitation characteristic of entire China. PVI was good at detecting the variability of precipitation since PVI can be applied to examine precipitation variability at flexible intervals (hourly, daily, weekly, or longer) based on the study goals.

Overall, the spatio-temporal analysis of precipitation occurrence frequency, mean precipitation depth, total precipitation and PVI shows that precipitation frequency reduced significantly, precipitation intensity increased significantly, total precipitation increased insignificantly, and the pattern of precipitation depicted a uniform trend in China under climate change. A combination analysis of multiple indicators should be adopted regionally and seasonally in order to have an exhaustive view on the spatio-temporal variation in China. Specifically, PVI showed a significant trend of increase in spring (Fig. 3e) while a significant decreasing trend in summer (Fig. 3f) in Yangtze Plain, which mean the precipitation pattern has a sporadic trend in spring, but a uniform trend in summer there. If we related this result to the trend of precipitation occurrence frequency there, we can find out that there is a downward trend of precipitation occurrence frequency in spring (Fig. 1e) and an upward trend of precipitation occurrence frequency in summer (Fig. 1f). The Yangtze Plain is the main Rice-production Areas of China. The sporadic with decreasing frequency precipitation trend in spring there would greatly affect the cultivation of rice seedlings and spring ploughing, and the uniform with increasing precipitation frequency trend in summer would increase the risk of flooding there. In autumn of the north-eastern Inner Mongolia and the northeast region, there is a uniform with decreasing frequency trend of precipitation as PVI showed a significant negative trend (Fig. 3g) and the precipitation occurrence frequency showed a significant decreasing trend (Fig. 1g), which would increase the drought risk there. In the southwest China, the total precipitation showed a significant decreasing trend (−1.54 mm/yr). There is a significant upward trend of PVI in winter (Fig. 3h) and summer (Fig. 3f). Combining the result of other indices, there is a sporadic with decreasing frequency (Fig. 1f) and intensity (Fig. 2f) trend of precipitation, and the total precipitation (Fig. 4f) reduced rapidly in summer (−0.72 mm/yr) there. In winter, there is a sporadic with decreasing frequency (Fig. 1h) and increasing intensity (Fig. 2h) trend of precipitation. Though there is little change of total precipitation (0.04 mm/yr) in winter for the past 55 years, the sporadic and concentrated trend of precipitation is also an important factor caused the drought there increasing frequently, which bring drinking water problems for people and animals in winter recent year. The total precipitation showed a significant decreasing trend in South-West region (−1.54 mm/yr). In northwest, the precipitation frequency, intensity and total precipitation increased significantly, and PVI depicted a decreased trend. Northwest is an arid water-deficient region, the uniform with increasing precipitation frequency and intensity trend is beneficial for the vegetation and agriculture production there. The driving force to these changes may related to the increased frequency of ENSO, which could make extreme precipitation more likely in China^38^.

This study can help us understand changing precipitation processes in China which may affect flood, drought, plant growth, and rice yield in China. These changes would also have significant influence on the hydrological process. By giving higher spatial resolution information of precipitation indicators, this study can help researchers and government better understand the cause of flooding or drought, and make more reasonable associated water resources management plans. Policymakers should take into account the variability of precipitation under global climate change when making relative policy to guide the development of agriculture. As a lot of other countries experience similar climate types and environments to China in the world, this study would provide constructive information such as agricultural management, water resources management, and related adaptation strategies for them.

## Method

### Data sets

**Figure 8:**
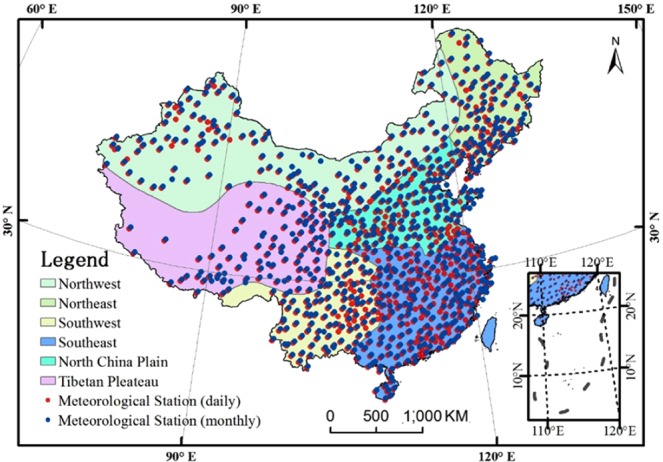
Distribution of the meteorological stations in China.

Daily (monthly) precipitation data of 819 (756) meteorological stations were acquired from the China Meteorological Administration (http://data.cma.cn/) for the period 1951–2015. The precipitation stations were distributed relatively evenly in space although a little sparse in the northwest (NW) regions and Qinghai-Tibet Plateau (QP) of China (Fig. 8).

Quality control was conducted to ensure a suitable dataset for the study. We kept the correct record, removed the low quality data (suspicious and incorrect record) using the quality controlled code included in the CMA precipitation observation datasets. A simple data integrity statistic was applied on these data through the calculation for the percentage of operational stations (the count of operational stations divided by the total amount of stations). Because instruments malfunctioned in the preliminary years when the majority of meteorological stations across China were initially formed, there are frequent lapses in the time series before 1960 (the percentage of operational stations >87% for monthly precipitation data and the percentage of operational stations >94% for daily precipitation data after 1961); accordingly, we eliminated those records in the early years from this evaluation and depended solely on data documented between 1961–2015. With respect to the quality control of missing data, the amount of missing data was less than 10% for each of the selected stations^39^; thus, the absent values were disregarded in the analyses below. Each of the precipitation datasets used in the study passed quality control standards^29^. Eventually the proper dataset is selected by excluding stations on the islands of South China Sea, which are under a maritime climate system, from the quality controlled data. The daily (monthly) precipitation time-series from 818 (754) meteorological stations were interpolated to grid precipitation surfaces (spatial resolution 5 km × 5 km) from 1961 to 2015.

The spatio-temporal assessment of precipitation variability was based on the calculation of both interpolated grid surfaces and original station observations for supplementary verification. For the analysis based on the calculation of the observations, to avoid bias in the trend analysis owing to absent data from the stations, only stations with fewer than 31 days or one month of yearly missing data were selected for annual (676 stations for daily precipitation and 545 stations for monthly precipitation) trend analysis. The trend analysis in the dry and wet seasons used the same rules in selecting study stations. For seasonal data, stations with more than 88 days for daily precipitation or equal to three months of yearly valid data for monthly precipitation were selected for trend analysis.

**Figure 9:**
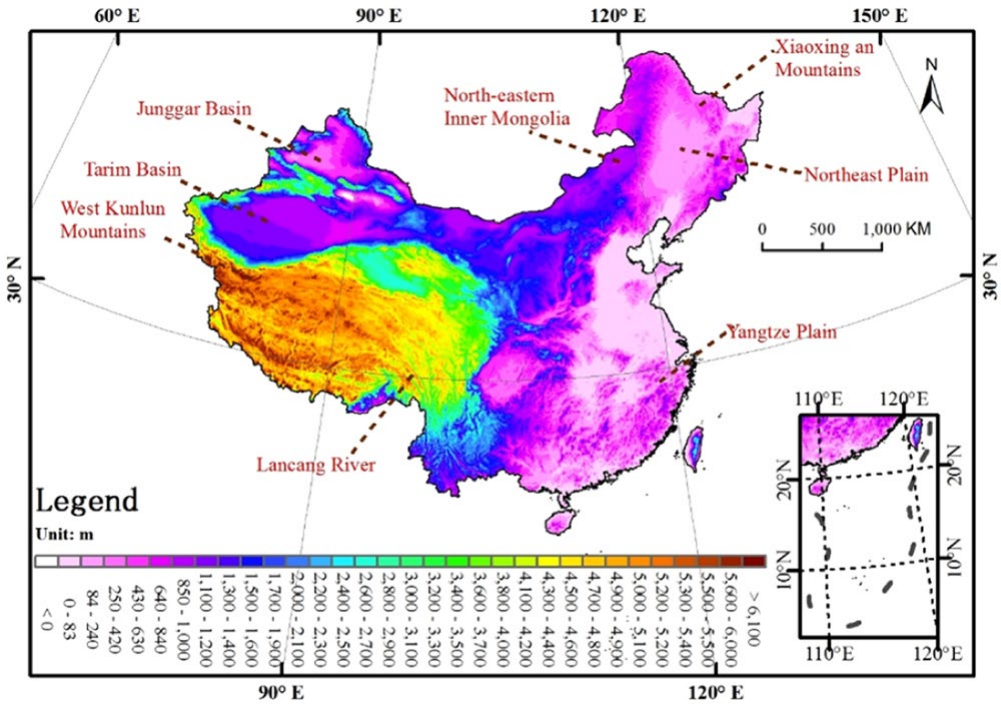
Rebuilt 5-km resolution DEM for China. The map was generated by the software ArcGIS 10.1.

Precipitation is strongly impacted by topography. The digital elevation model (DEM) was used as the independent covariate in generating the precipitation grid surface. Hutchinson (1998) reported that the precision of precipitation interpolation may be significantly increased with a appropriate DEM^40–42^. The DEM adopted in this evaluation initiated from Global Multi-resolution Terrain Elevation Data 2010 (GMTED2010), which is the most recent and potentially the premier global terrain product thus far. This DEM is similar to an actual DEM instead of a top-of-canopy surface as located in certain satellite topography data products. As the sharp terrain features on the resulting climate surface grids are usually not true, a rebuilt 5-km resolution DEM suppresses these features and facilitates precipitation grids surface with more realistic spatial distributions. The DEM in this study is a DEM (Fig. 9) rebuilt from the GMTED2010 product (spatial resolution of 7.5-arc-second) with ANUDEM5.3^43^.

### Generation of precipitation grid surfaces

As the station-based observation data was spatially patchy, interpolation was used to expand the station-based observation data to a daily grid precipitation surface. ANUSPLIN was applied to generate the precipitation grid surfaces for China. This method is an integration and generalization of multivariate linear regression. ANUSPLIN is a spatial interpolation program for meteorological data which has been implemented in a lot of researches^14,26,44^.

The ANUSPLIN is created with an algorithm called thin plate smoothing splines, which make it suitable for interpolating climate data with large amounts of noise. The noisy multivariate climate data are treat as a function with one or more independent variables while fitting a climate surface.

The partial thin-plate smoothing spline model for the predicted value *z*_*i*_ at location *x*_*i*_ is shown as follows:

1 $${{\rm{z}}}_{i}=f({x}_{i})+{\sum }_{j=1}^{p}{\beta }_{j}{\varPsi }_{j}({x}_{i})+{\varepsilon }_{i}\,(i=1,\ldots ,n;j=1,\ldots ,p)$$

where *n* is the number of observational data, *f* represents a smoothing function which needs to be estimated, *β*_*j*_ denotes a series of parameters (*p* dimensions) which also needs to be estimated, ${\varPsi }_{j}$ are a series of function (*p* dimensions) of independent variables, and the *ε*_*i*_ represent independent, random, and zero mean errors. More details about ANUSPLIN can be found in ANUSPLIN user guide^11^ and related research references^40,45^.

In this study, ANUSPLIN4.4^11^ was measured value at position *i*,used to interpolate precipitation as a function of horizontal position (latitude and longitude) and vertical position (elevation). The generated precipitation grids in this study were merged with a DEM with the same resolution, which had a cell size of 5 km × 5 km (0.05 degree).

### Performance evaluation criteria of ANUSPLIN validation

The interpolated precipitation grid surface may contain deviations arising from the process of interpolation, which is one of the main uncertainty source for precipitation spatio-temporal variability analysis. The ANUSPLIN enabled a robust detection of error using cross validation statistics that were applied to evaluate the overall error of the interpolated spline grid presented in this study. For each interpolation, the ANUSPLIN performs cross-validation by implicitly holding out each station in turn and calculating the individual deviation between the measured value and the fitted surface value at that station’s location^11^.We adopted cross-validation statistics to evaluate the overall error of the interpolated precipitation grid. The mean error (ME), root mean square error (RMSE), mean absolute error (MAE), and relative root mean square error (RRMSE) were selected as interpolation performance evaluation criteria in this study. These methods are described below^46^.

2 $${\rm{ME}}=\frac{1}{n}{\sum }_{i=1}^{n}[ob{s}_{i}-si{m}_{i}]$$

3 $${\rm{RMSE}}=\sqrt{\frac{1}{n}{\sum }_{i=1}^{n}{[ob{s}_{i}-si{m}_{i}]}^{2}}$$

4 $${\rm{MAE}}=\frac{1}{n}{\sum }_{i=1}^{n}[|ob{s}_{i}-si{m}_{i}|]$$

5 $${\rm{RRMSE}}=\frac{RMSE}{ob{s}_{i}}\times 100 \% $$

where *obs*_*i*_ is the measured value at position *i*, *sim*_*i*_ denotes the predicted value at position *i*, and *n* is the amount of samples. The RRMSE is defined as the RMSE divided by the daily observed precipitation at the cross-validation station. A value of zero for ME, MAE, RMSE, and RRMSE depicts an accurate match between the measured and predicted value.

### Precipitation indicators

We here investigated five precipitation indices—precipitation occurrence frequency, mean precipitation depth, precipitation variability index and total precipitation in China. These precipitation indices are defined as follows.

### Precipitation occurrence frequency (λ_0_)

The precipitation occurrence rate describes the number of rainfall events within a certain period of time. In this study, this period was confined to one year or one season and the precipitation occurrence rate was the number of rainfall days within one year or season.

6 $$\lambda =\sum r,$$

where

7 $$r=\{\begin{array}{c}\,0\,if\,{p}_{i}=0\\ \,1\,if\,{p}_{i} > 0\end{array}.$$

Here, *p*_*i*_ is the measurement of daily precipitation on day *i* and *i* = 1, …, n is the sequential count of days in that period. Precipitation occurrence frequency (λ_0_) is defined as the precipitation occurrence rate (λ) divide by the number of days in that period.

### Mean precipitation depth (1/θ, unit: cm)

The mean precipitation depth depicts the mean precipitation intensity at a single time^47^.

8 $$\frac{1}{\theta }=\frac{TP}{10\lambda }$$

Here, TP is total precipitation (as described later), λ is the precipitation occurrence rate.

In this study, this period was confined to a year or a season and the mean precipitation depth was total amount of precipitation divided by the precipitation occurrence rate within that period.

### Precipitation variability index (PVI)

The PVI index has recently been introduced as an effective way to evaluate the impacts of drought on tree mortality^48^. The unit is dimensionless, and the PVI is calculated for each grid point according to the following:

9 $${\rm{PVI}}=\sqrt{\frac{{\sum }_{i=1}^{n}{({R}_{i}-\bar{R})}^{2}}{n}}$$

Where

10 $${R}_{i}=\frac{{C}_{i}}{{E}_{i}}$$

11 $$\bar{R}=\frac{{\sum }_{i=1}^{n}{R}_{i}}{n}$$

12 $${C}_{i}={\sum }_{j=1}^{i}{p}_{j}$$

13 $${E}_{i}=i\bar{p}$$

Here, {*p*_1_, *p*_2_, …, *p*_*n*_} represents a set of observed data for precipitation time series and *i* = 1, …, n. The values of *p*_*i*_, *i* = 1, …, *n* are supposed to be observed at even intervals. Based on the proposed use of the PVI, the intervals can be defined as hourly, daily, weekly, or longer. We assumed that the values of *p*_*i*_ were observed daily precipitation with units of millimetres for this evaluation. The dimensionless PVI indicator depicts precisely consistent precipitation with a value of zero and increases as precipitation events get more infrequent.

### Total precipitation (TP, unit: mm)

Total precipitation is calculated through the accumulation of daily precipitation data within a period.

14 $${\rm{TP}}={\sum }_{i=1}^{n}{p}_{i}$$

Here, *p*_*i*_ is the measurements of daily precipitation in day *i* and *i* = 1… n is the sequence count of days in that period. It is widely used in studies related to precipitation that reflect the total amount of precipitation in a period.

### Mann–kendall trend analysis and simple linear regression

A Mann–Kendall (MK)^49^ trend analysis and simple linear regression was conducted to examine long-term trend as well as significant trend regions for these indicators based on precipitation indicator surfaces.

The MK trend analysis is a widely used non-parametric way in assessing the trend and detecting the breakpoint of long-term meteorological or hydrological time series and it is greatly endorsed by the World Meteorological Organization for relative studies^50,51^. More details about MK trend analysis can be found in related research references^52,53^.

The simple linear regression is a type of commonly used parametric statistical method for measuring the relationship between the independent input variable (in this case, the time) and the dependent target variable (in this case, precipitation occurrence frequency, mean precipitation depth, PVI, or total precipitation). The method is based on several hypotheses such as the linear relationship between the input variable and the target variable, the input or target variable are homoscedastic and normally distributed^54^.

## Data Availability

The datasets produced throughout and/or analysed throughout the current evaluation are available from the corresponding author on reasonable request.
